# Tuning spatially fractionated radiotherapy dose profiles using the moiré effect

**DOI:** 10.1038/s41598-024-55104-7

**Published:** 2024-04-11

**Authors:** Fardous Reaz, Erik Traneus, Niels Bassler

**Affiliations:** 1 Department of Clinical Medicine, Aarhus University, Aarhus, Denmark; 2 Danish Centre for Particle Therapy, Aarhus University Hospital, Aarhus, Denmark; 3 RaySearch Laboratories AB, Stockholm, Sweden

**Keywords:** Radiotherapy, Radiotherapy, Radiotherapy, Radiotherapy

## Abstract

Spatially Fractionated Radiotherapy (SFRT) has demonstrated promising potential in cancer treatment, combining the advantages of reduced post-radiation effects and enhanced local control rates. Within this paradigm, proton minibeam radiotherapy (pMBRT) was suggested as a new treatment modality, possibly producing superior normal tissue sparing to conventional proton therapy, leading to improvements in patient outcomes. However, an effective and convenient beam generation method for pMBRT, capable of implementing various optimum dose profiles, is essential for its real-world application. Our study investigates the potential of utilizing the moiré effect in a dual collimator system (DCS) to generate pMBRT dose profiles with the flexibility to modify the center-to-center distance (CTC) of the dose distribution in a technically simple way.

We employ the Geant4 Monte Carlo simulations tool to demonstrate that the angle between the two collimators of a DCS can significantly impact the dose profile. Varying the DCS angle from 10^∘^ to 50^∘^ we could cover CTC ranging from 11.8 mm to 2.4 mm, respectively. Further investigations reveal the substantial influence of the multi-slit collimator’s (MSC) physical parameters on the spatially fractionated dose profile, such as period (CTC), throughput, and spacing between MSCs. These findings highlight opportunities for precision dose profile adjustments tailored to specific clinical scenarios.

The DCS capacity for rapid angle adjustments during the energy transition stages of a spot scanning system can facilitate dynamic alterations in the irradiation profile, enhancing dose contrast in normal tissues. Furthermore, its unique attribute of spatially fractionated doses in both lateral directions could potentially improve normal tissue sparing by minimizing irradiated volume. Beyond the realm of pMBRT, the dual MSC system exhibits remarkable versatility, showing compatibility with different types of beams (X-rays and electrons) and applicability across various SFRT modalities.

Our study illuminates the dual MSC system’s potential as an efficient and adaptable tool in the refinement of pMBRT techniques. By enabling meticulous control over irradiation profiles, this system may expedite advancements in clinical and experimental applications, thereby contributing to the evolution of SFRT strategies.

## Introduction

External beam radiotherapy (EBRT) is a widely used treatment modality for various types of cancer that has shown remarkable curative and palliative results for patients^1^. The major drawback of this approach, however, is that it invariably irradiates normal tissue, which limits the effectiveness of EBRT in many cases, especially in the treatment of radioresistant tumors. Irradiation of healthy tissues can cause severe complications that can prevent optimal therapeutic efficacy. To overcome this critical challenge and improve the safety and effectiveness of EBRT, spatially fractionated radiotherapy (SFRT) was introduced by Alban Köhler at the beginning of the twentieth century^2^. Köhler utilized metal grids to implement the SFRT. Initially, using orthovoltage X-rays, SFRT was employed to treat deep-seated and bulky tumors to reduce skin toxicities. However, the development of megavoltage X-rays in the 1950s offered the advantage of reduced surface doses for deep-seated targets, rendering SFRT obsolete at that time. SFRT has resurfaced as a possible treatment approach in recent decades. The renewed interest in SFRT is due to its potential to complement conventional homogeneous dose distributions by reducing the risk of severe post-radiation effects and tumor regression while improving local control rates^3–7^.

SFRT uses an inhomogeneous dose distribution in healthy tissue to reduce radiation-induced damage. In addition, the clinical benefits of heterogeneous tumor doses are also reported^8–14^. Instead of a wide and laterally homogeneous field of conventional radiotherapy, SFRT uses several beamlets with suitable spacing to produce sharp lateral dose gradients in a periodic pattern of high and low doses, referred to as “dose-peaks” and “dose-valleys”. The distance between two consecutive peaks (or valleys), called center-to-center distance^15^ (CTC), is a crucial parameter that can characterize the SFRT dose distribution. Based on beam size and interbeam spacing, SFRT is classified mainly into four groups: GRID radiotherapy, LATTICE radiotherapy, Minibeam radiotherapy (MBRT), and Microbeam radiotherapy (MRT)^16^. The biological basis of SFRT is not fully understood yet; however, it is speculated to be related to multiple effects, such as dose-volume effect, radiation-induced bystander effects, microvascular alterations, and immunomodulation^10,17–21^.

Over the past two decades, proton therapy has emerged as a promising modality that can save healthy tissues because of its favorable depth-dose profile compared to conventional photon therapy. Proton therapy can potentially reduce the integral dose to healthy tissue by a factor of 2–3^22^. SFRT through the application of proton minibeam radiotherapy (pMBRT) has garnered interest as a way to further increase the therapeutic window in proton therapy^23^. This treatment modality uses proton beams of size around 1 mm with a few millimeters spacing. Several studies have demonstrated the advantages of pMBRT in complementing conventional proton therapy, highlighting its potential to improve clinical outcomes ^23–28^.

To effectively implement pMBRT, a desired irradiation profile can be obtained using a multi-slit collimator^29,30^, which consists of a metal block with a series of apertures inserted into the beamline. Moreover, an inhomogeneous dose distribution of SFRT can also be administered using alternative methods such as the multi-leaf collimator^31^ and the scanning dynamic collimator^32^. In addition to mechanical collimators, a magnetically shaped beam can be utilized to enhance the dose contrast with a reduced number of neutron production^33,34^. However, considering the limitations of existing proton therapy facilities and the technological complexity, a static mechanical collimator seems to be a simple solution at the present stage, which readily can be implemented at a proton therapy research facility. The optimal dose layout for pMBRT is contingent upon factors like tumor dimension, contour, and depth, rendering it inherently diverse. Furthermore, achieving a uniform dose distribution across the tumor is intricately influenced by the dimensions of the beam and the spacing between adjacent beams, factors critical to the precision of treatment delivery^35^. A limitation arises from the fact that a singular collimator cannot cater to variable CTC requirements. Crafting patient-centric collimators can resolve this, but the associated demands in resources, time, and expenditure can be daunting. In this study, we present a novel method to achieve variable CTC by employing two MSCs grounded in the principles of the moiré effect^36,37^.

**Figure 1:**
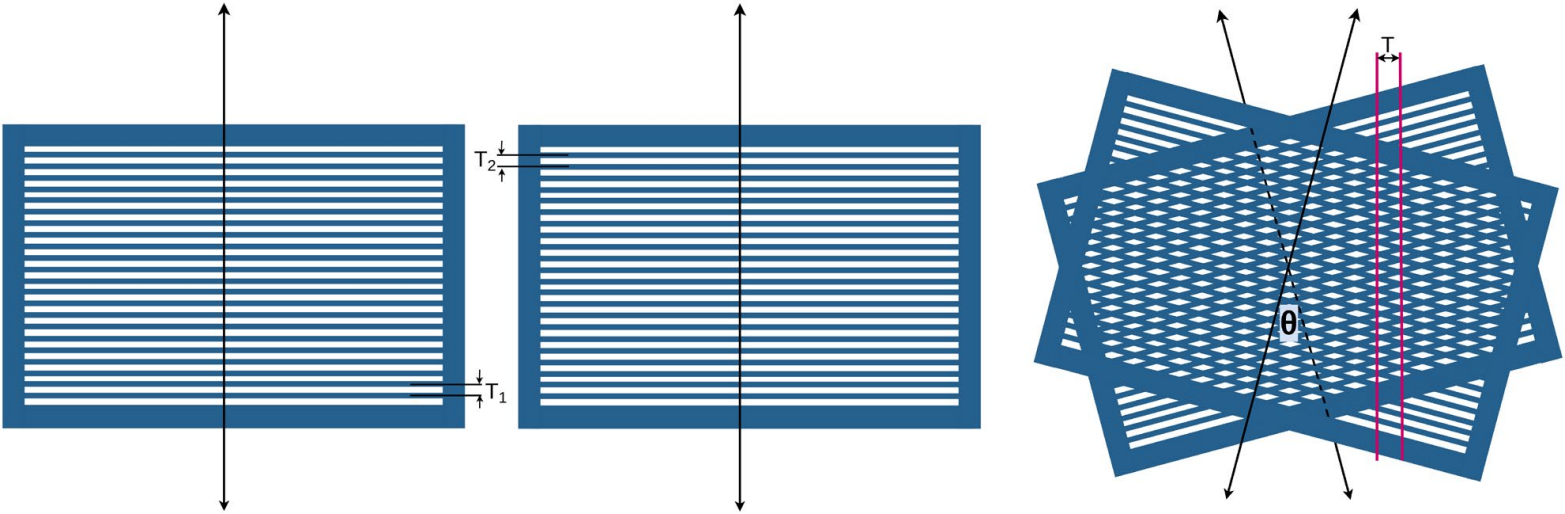
Visual representation of the moiré effect in a dual MSC system. The angle between the collimators ($\theta$) can alter the resulting period (T) of the system. $\mathrm {T_1}$ and $\mathrm {T_2}$ represent the period (CTC) of the individual MSCs.

MSCs are typically characterized by their regular periodic structures, comprising alternating apertures and septa. The distance measured from the center of one aperture or septum to the center of the next constitutes the ’period’ (equal to the geometrical CTC^15^) of an MSC, as indicated by $T_1$ and $T_2$ in Fig. 1. The throughput of a collimator is quantified as the ratio of the collimator’s total open area to its overall surface area^15^. Therefore, the defining attributes of an MSC can be succinctly represented by its period and throughput.

The moiré effect is an intriguing visual phenomenon that occurs when two transparent patterns with regularly spaced structures overlap. Specifically, it is triggered when two sets of lines or grids are superimposed at a certain angle or with a slight shift. This superimposition results in the formation of a secondary pattern, termed a moiré fringe^38–41^. When the grids move over each other, they create a new pattern of moving waves or bands that is not present in each individual grid^42^. The existence of this is due to the constructive and destructive interference of the overlapping structures. Notably, the emergent pattern of intensity exhibits a periodic nature. This period is contingent on the period of the individual grids and their relative orientation, demonstrating the intricate and dynamic nature of the moiré effect.

Within the captivating scope of the moiré effect, consider two collimators characterized by periods $T_1$ and $T_2$, respectively. When these collimators are superposed at an angle $\theta$ relative to each other, the resulting collimating pattern acquires a distinct period *T*. The period of the observed moiré patterns can be computed using the following equation, which considers the periods of the individual collimators and their relative angle of orientation^43^.

1 $$\begin{aligned} \ T = \frac{T_1}{\sqrt{1 - 2\frac{T_1}{T_2}~cos\theta + \left( \frac{T_1}{T_2}\right) ^2 }}, \end{aligned}$$

The moiré patterns arise from the intricate interactions between the structural elements of the radiation collimators. These patterns can be manipulated by altering the angle between the collimators of a dual collimator system (DCS), offering a degree of control over the resultant pattern. This study delves into the effects of several MSC’s parameters of a DCS on proton beams to deliver spatially fractionated doses. Our goal is to demonstrate the potential of utilizing the moiré effect in developing a DCS that is capable of producing adjustable CTC for SFRT.

## Methods

**Figure 2:**
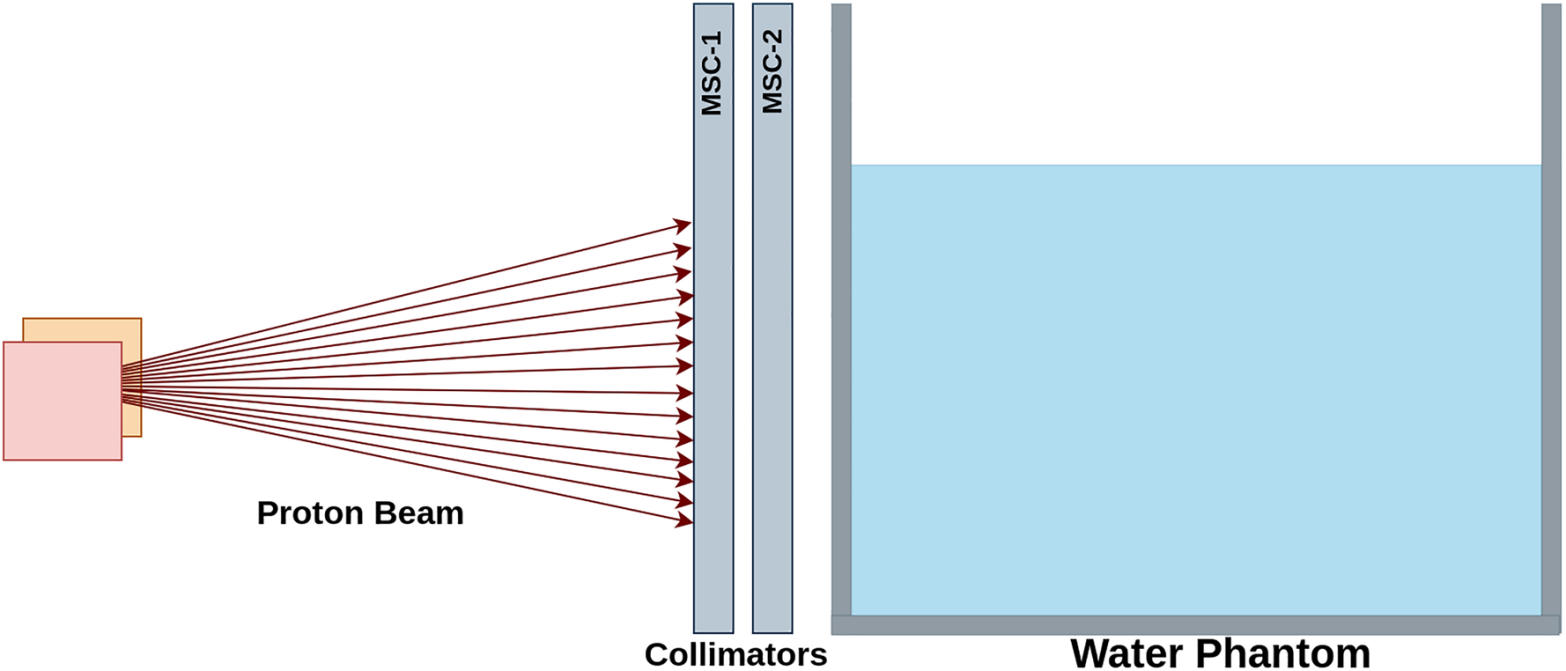
Outline of the simulated setup.

Utilizing the Geant4 Monte Carlo (MC) simulation tools, we explored how different parameters of a dual MSC system could affect the resulting spatially fractionated dose profile. Foreseeing future experimental verification of our findings at the Danish Centre for Particle Therapy, Aarhus, Denmark (DCPT), we replicated an established experimental setup for mouse studies within our simulations^44–46^. Since active scanning methods, also known as pencil beam scanning (PBS), are commonly used in modern proton therapy centers, we simulated a PBS system-based treatment plan in our studies. Figure 2 visually represents the geometric arrangement of the simulations. We opted for tungsten as the collimator material, which was adopted from the Geant4 database^47^. This choice was made due to the high density of tungsten, which facilitates a compact DCS design.

The water phantom was simulated with dimensions 45 cm $\times$ 24 cm $\times$ 34.55 cm with 1.0 cm polymethyl methacrylate (PMMA) wall thickness and filled with water with a density of 1 $\mathrm {g/cm^3}$. The design is based on the mouse phantom used for radiobiological studies^46^.

### Beam modeling

The beam model for the MC simulations emulates the beam parameters of the Varian ProBeam PBS system installed at DCPT. The beam model provides the spot size, divergence, covariance, and actual energy for each nominal energy layer, and is openly available^48^. It was originally prepared by TOPAS^49^ MC simulations and validated against experimental data obtained in DCPT treatment rooms. Together with the beam deflection and the iso-center spot positions provided by the treatment plan, the phase-space for primary particles is generated at a well-defined plane prior to the collimator.

The energy-dependent sigma ($\sigma _u$), divergence ($\sigma _{u'})$, and covariance ($\sigma _{uu'}$) for each beam were utilized to construct the bivariate Gaussian phase-space of the spatial (*u*) and angular ($u'$) distribution^50^. The correlation coefficient ($\rho$) between spatial and angular distribution were calculated as

2 $$\begin{aligned} \rho (u, u') = \frac{\sigma _{uu'}}{\sigma _{u} \sigma _{u'}} \,. \end{aligned}$$

With Gaussian distributed random numbers ($Z_u$ and $Z_{u'}$), the spatial and angular distribution was constructed (with the mean values of $\mu _u$ and $\mu _{u'}$) from the relation

3 $$\begin{aligned} u= & {} \sigma _{u}Z_u + \mu _u, \end{aligned}$$

4 $$\begin{aligned} u'= & {} \sigma _{u'}\left( \rho Z_u + \sqrt{(1 - \rho ^2)}~Z_{u'} \right) + \mu _{u'}. \end{aligned}$$

### Treatment plan

The Eclipse^51^ treatment planning system was used to develop a plan to deliver uniform doses at the planning target volume (PTV) for non-collimated irradiation. The dimension of the PTV is based on the previously described in vivo mouse setup, and was 100 mm $\times$ 25 mm $\times$ 30 mm within $x_{\textrm{min}} = -50~\textrm{mm}$ to $x_{\textrm{max}} = 50~\textrm{mm}$ (horizontal), $y_{\textrm{min}} = -12.5~\textrm{mm}$ to $y_{\textrm{max}} = 12.5~\textrm{mm}$ (vertical), and $z_{\textrm{min}} = 55~\textrm{mm}$ to $z_{\textrm{max}} = 85~\textrm{mm}$. The z-positions are positive along the beam axis, and relative to the isocenter plane, where the surface of the phantom was positioned. The plan comprised of eight energy layers ranging from 84.7 MeV to 107.6 MeV with an energy spread of 1.09% to 1.21% (1 sigma) to ensure longitudinal uniformity^46^.

### MC simulation setup

All the results presented in this work were simulated using Geant4 ^52^ version 11.3. We implemented a pre-built reference physics list, which is activated by including “QGSP_BIC_HP_EMY”. Our preference was based on several published recommendations to optimize the accuracy and the computation time of hadron therapy simulation ^53–55^. It initializes Quark–Gluon String Precompound (QGSP) that defines the models for inelastic hadron-nucleus interactions ^56^. The Binary cascade model is applied for neutrons and protons between 0 and 6 GeV. The precompound model includes the Fermi breakup, neutron, light ion, and photon evaporation models. Inelastic nucleus-nucleus scattering is governed by the Binary Light Ion Cascade (BIC) within the energy range of 0 and 6 GeV/nucleon.

The maximum step length was tuned to 0.2 mm for all particles in the water phantom, including the PMMA container, to improve the simulation accuracy with a feasible execution time. In the rest of the geometry, we used the default step length. The production range cut was set to 10 $\mathrm {\mu m}$ for photon, which is equivalent to 990 eV in water. For electrons and protons, it was set to 50 $\mathrm {\mu m}$, with corresponding energy of 57.33 keV and 5.0 keV in water, respectively.

### Dose computation

The entrance dose was scored between 0 to 4 mm along the beam axis in the water phantom (including the wall of the container), and the spread-out bragg peak (SOBP) dose was recorded at a depth from 68 to 72 mm. A bin depth of 4 mm was chosen to improve the MC statistics. Laterally, higher resolutions are used where spatial fractionations occur. Along the transverse plan, an area of $150~\textrm{mm} \times 100~\textrm{mm}$ was included with its origin in the isocenter. The entire volume was comprised of $1500 \times 1000 \times 1$ voxels. The size of each scoring voxel was $0.1 \times 0.1 \times 4.0 ~\textrm{mm}^3$. We estimated the dose in each voxel of volume *V* as

5 $$\begin{aligned} D = \frac{1}{\rho V} \sum _{i=1}^{n} \delta E_i, \end{aligned}$$

Where $\delta E_i$ represents the energy deposited in the $i^{\textrm{th}}$ instance within a series of *n* total energy depositions that occur within a volume V, which is occupied by a material of density $\rho$. The dose distribution was used to calculate the full width at half maximum (FWHM) of the beam along the x- and y-directions at the field’s center.

## Results

We have studied the impact of different parameters of a dual MSC system on spatial fractionated dose profiles.

### Impact of angle

**Figure 3:**
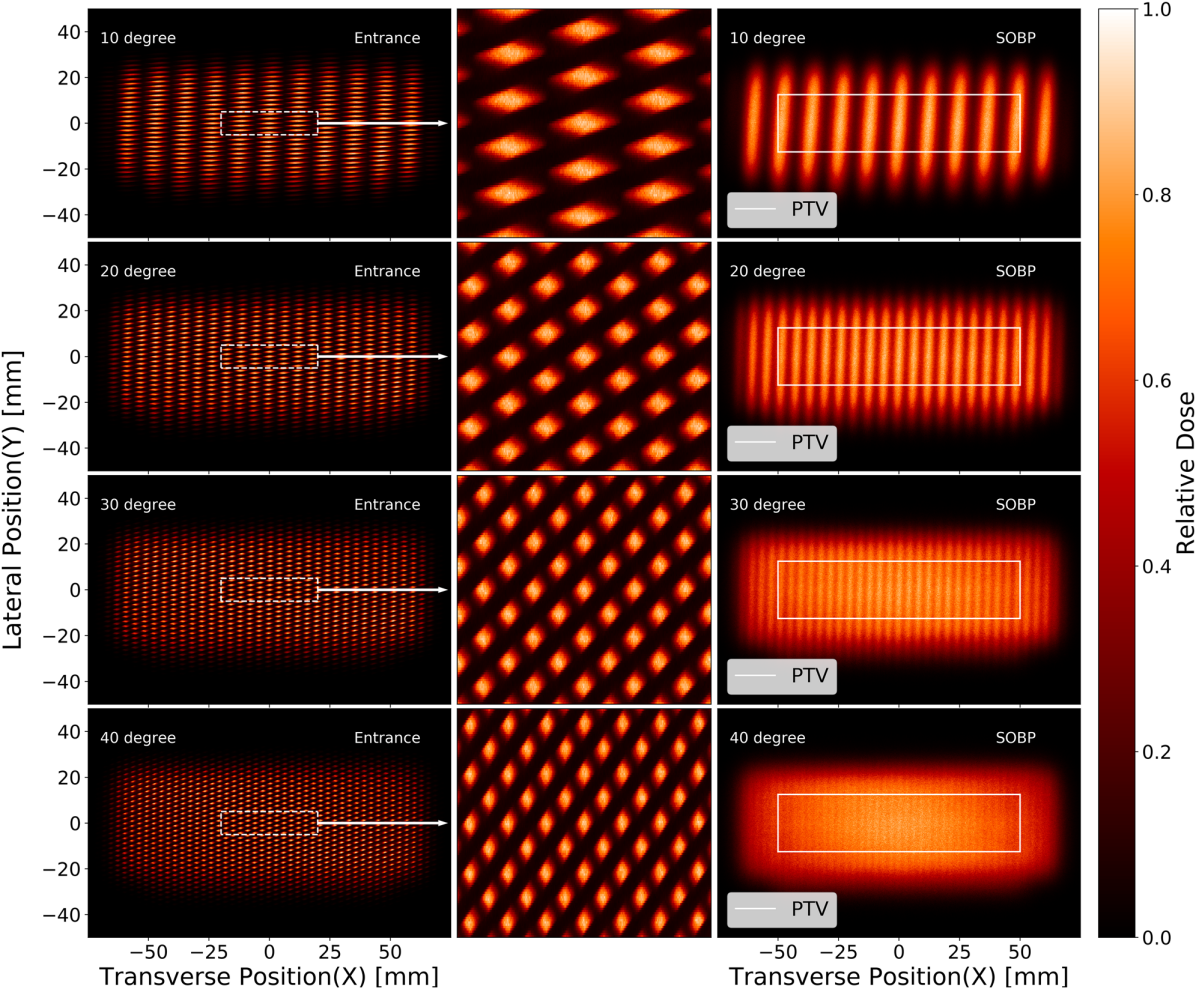
Simulated spatially fractionated dose patterns (moiré patterns) created by a DCS at varying angles. The middle column displays the central portion of the field, demarcated by a white line in the left column, on an enlarged scale, though the original aspect ratio is not preserved. The white box in the left panel marks the planning target volume. Notice that variations in the angle notably modulate the beam’s shape and size, highlighting the complex interplay between angular adjustment and beam geometry.

To assess the influence of the angle between two collimators of a DCS, we conducted simulations using two identical MSCs, each with 2 mm period (CTC of MSC) and 50% throughput (1 mm gap with 1 mm septal thickness). Each collimator has 30 mm thickness, which is sufficient to stop the proton beam of the simulated energy range. The beam exit side of the collimator (MSC-2 in Fig. 2, placed downstream) was positioned 5 mm from the phantom’s surface. To achieve a sharp dose contrast, we keep a minimal distance between the collimator and the phantom. With a consistent 5 mm gap between the two MSCs, we noticed a significant impact of the DCS angle on the dose distribution as depicted in Fig. 3. The variation in angle alters the transverse dose profiles at the entrance of the phantom and the center of the SOBP (68 mm to 72 mm depth, within PTV). Interference between two MSCs of the DCS causes a spatial dose fractionation effect in both transverse (x and y) directions.

The DCS showed a promising ability to function as a 2D grid, dividing the extensive radiation field into various parallel beamlets. These beamlets’ size was directly influenced by the relative orientation of the collimators, along with their specific parameters, such as aperture size and period. For a given set of collimators, the size of the beamlets depends solely on the angle. By tuning the angle between the collimators, we observed precise control over the beamlet size, leading to a consequential impact on the CTC of the dose distribution. This level of control presented exciting possibilities for tailoring CTC for various patient-specific SFRT treatment modalities.

With an increase in the angle between the two MSCs, the spacing between the high-dose segments along the y-direction remained consistent at the entrance of the phantom. However, as the particles encountered multiple Coulomb scattering MCS) in water, the beamlets experienced broadening, causing the y-spacings to gradually disappear in the center of the SOBP. Simultaneously with the variation of angle, we observed an alteration in the intriguing periodic moiré patterns along the x-direction. As the angle between the collimators progressively increased, the period of these moiré dose patterns demonstrated a gradual decrease. This finding offered strong support for the Eq. (1), which describes the relationship between the collimator angle and the resulting period of the moiré pattern.

Moreover, the inverse relationship between the angle and the moiré period is mirrored in the beam size, with larger angles producing more refined and narrower beamlets (Table 1). It should be noted that along the y-direction, the beam size remains relatively constant, showing minimal sensitivity to changes in angle. However, in the x-direction, where the moiré period is manifested, the beam size exhibits a marked decrease as the angle widens.

We investigated the effects of five different angles (10^∘^, 20^∘^, 30^∘^, 40^∘^ and 50^∘^) of DCS on the dose profile. Our primary focus was on the CTC, which is considered one of the most crucial parameters in characterization of the dose distribution of slit-collimated SFRT. We observed a clear trend of decreasing CTC values at the entrance of the phantom as we increased the angle between the two collimators. This decrease in CTC can lead to a more uniform lateral dose distribution throughout the PTV (cf. Fig. 3 bottom right image), a desirable outcome in conventional radiation therapy. As can be expected from the analytical formula (eq. 1), the difference between the CTC values at 30^∘^ and 40^∘^ angles appeared less pronounced than the changes observed at smaller angles as this is not really surprising since the cosine is not a linear function of angle. This suggests that beyond a certain angle, the alteration in the dose distribution becomes less significant with increasing angle.

Strategic selection of appropriate angles can offer the possibility of achieving a heterogeneous dose distribution at the PTV, which may be preferred for clinical adaptation, e.g., lattice therapy; However, obtaining tumor control with spatially fractionated i.e., heterogenous dose distributions is still under investigation. Conversely, in a PBS system, fine-tuning spot weight along with the angle of DCS can allow the attainment of a uniform dose at the PTV. This flexibility facilitates the delivery of customized dose distributions, tailoring them to precisely meet the specific needs of individual patients.

**Figure 4:**
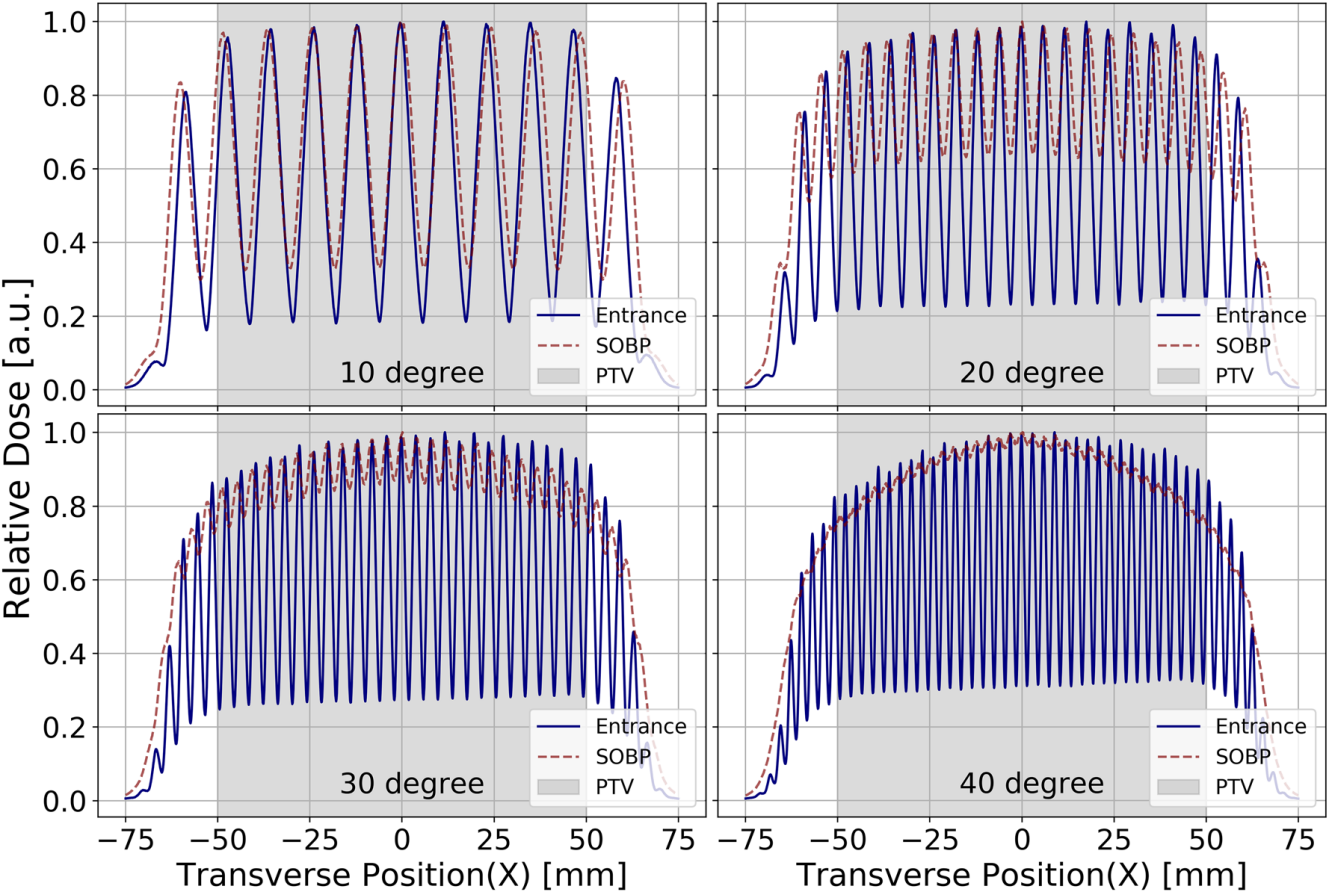
Lateral dose distributions at the entrance and the center of the SOBP along the morié fringes, as also shown in Fig.3.

Figure 4 presents the relative dose variation as a function of the lateral (x-direction) position at the entrance of the phantom for four different angles. The dose is normalized to the peak dose value for convenient comparison. A desirable dose contrast emerges between the peaks and valleys at the entrance. For the SFRT irradiation modality, a lower dose in the valleys is highly desirable in normal tissue. We observe that the valley dose is lowest when the angle between the collimators is the smallest, leading to the largest CTC. This outcome can be attributed to the fact that a smaller number of scattered protons can reach the shielded valley region. Additionally, the CTC of the dose distribution exhibits changes with the angle between the collimators. As the angle increases, more protons can reach the valley region, increasing the valley dose. This variation highlights the impact of angle selection on dose distribution and further emphasizes the importance of fine-tuning angles for optimal irradiation patterns.

In Table 1, we present a summary of the fractionated dose profiles obtained through calculations and simulations. To calculate the moiré period, we utilized the Eq. (1), which allowed us to quantify the periodic pattern generated by the interaction of two MSCs. Additionally, we determined the simulated period (CTC of dose) by calculating the average distance between the peaks. The percentage difference between the analytical and simulated moiré periods is assessed to obtain the deviation of the simulated result from the theoretical value. Furthermore, we determined the valley-to-peak dose ratio^15^ (VPDR) for each angle to quantify the dose contrast. With this parameter, we can measure the homogeneity of the dose distribution throughout the evaluated area. A lower VPDR value signifies a preferable sharp dose contrast at the phantom’s entrance.

**Table 1:** Comparative analysis of analytical and simulated period.

Angle (degree)	Analytical period / CTC (mm)	Simulated period / CTC (mm)	Difference (%)	VPDR	$\mathrm {FWHM_x (mm)}$	$\mathrm {FWHM_y (mm)}$
10	11.47	11.75	2.35	0.156	8.11	1.02
20	5.76	5.90	2.39	0.211	4.10	1.02
30	3.86	3.95	2.18	0.280	2.72	1.02
40	2.92	2.99	2.23	0.321	2.14	1.04
50	2.37	2.42	2.12	0.360	1.85	1.07

The table represents the variation in analytical and simulated moiré period, their relative difference, and VPDR as a function of the collimator angle at a period of 2 mm and a throughput of 50%. The simulated orientation of MSCs generated moiré pattern predominantly aligns along the x-direction.

The analytical moiré periods are compared to the Monte Carlo simulated periods and exhibit good agreement across all DSC angles, with percentage differences ranging from 2.12% to 2.39%. This finding serves as strong evidence of the consistency and reliability of our simulations in accurately capturing the periodic patterns generated by the moiré effect. Furthermore, the VPDR values obtained at the entrance of the phantom range from 0.156 to 0.360, indicating a favorable level of dose heterogeneity, which might be sufficient to observe the effect of SFRT. At larger angles, the VPDR increases, representing a reduced dose contrast.

### Impact of collimators period

The individual period of each MSC plays a crucial role in shaping the resulting dose profile of a DCS. We examined five distinct collimator periods (1.2, 1.6, 2.0, 2.4, and 2.8 mm) to understand their impact on both analytical and simulated moiré periods. For consistency, we assigned the same period to both MSCs within the DCS and analyzed the resulting dose profile to determine the CTC of the dose distribution. Simultaneously, using VPDR, we quantified the dose contrast for each period that corresponds to a specific angle. The data in Table 2 provides a comprehensive overview of the relationship between the MSCs’ period and the resulting spatially fractionated dose profile.

As the collimator period was increased, we observed corresponding increases in both the analytical and simulated moiré periods. Specifically, the simulated period exhibited a slightly greater increase than the analytical moiré period, resulting in a consistent percentage difference ranging from 2.11% to 2.24%.

When the collimator period was set at the smallest value of 1.2 mm, the VPDR reached its maximum at 0.433, indicating the lowest contrast between dose peaks and valleys. As the collimator period increased to 2.8 mm (largest), the VPDR gradually decreased to 0.197, signifying the highest level of dose inhomogeneity. This trend suggests that as the collimator period increases, more pronounced dose contrasts become attainable. Simultaneously, there is an enlargement in the beam size in both the x- and y-directions, a change that is directly tied to the increase in the collimator’s period (Table 2).

**Table 2:** Comparison between analytical and simulated period. The table represents the variation in analytical and simulated moiré period, their relative difference, and VPDR as a function of the collimator period at a collimator angle of 30 degree and a throughput of 50%.

Collimators’ period / CTC (mm)	Analytical period/CTC (mm)	Simulated period / CTC (mm)	Difference (%)	VPDR	$\mathrm {FWHM_x (mm)}$	$\mathrm {FWHM_y (mm)}$
1.2	2.31	2.37	2.15	0.433	1.67	0.64
1.6	3.10	3.16	2.18	0.343	2.07	0.81
2.0	3.86	3.95	2.18	0.280	2.72	1.02
2.4	4.64	4.74	2.11	0.225	3.47	1.19
2.8	5.41	5.53	2.24	0.197	4.06	1.36

### Impact of collimators’ throughput

We explored the impact of the variation of the collimator throughput on the dose profile while maintaining a constant period of each MSC of the DCS. This approach affected only the aperture size, which influenced the percentage of primary protons transmitted through the collimation system. We performed simulations using collimators with a 2 mm period and adjusted the throughput from 30% to 70% to evaluate the consequent alterations in the dose profile.

Since the parameters of the moiré period remained static (Eq. 1), we anticipated a stable CTC at the entrance of the phantom. The outcomes of the simulations yielded CTC values within the range of 3.91 mm to 3.97 mm for the assessed throughputs. These results show a minimal deviation from the theoretically computed value of 3.86 mm, with a discrepancy of merely 2.1%. These data affirm the simulation’s effectiveness in upholding the theoretical formulation. Furthermore, an increment in throughput not only results in a higher fraction of primary protons propagating the collimators but also proportionally enlarges the beam size and the CTC of the dose profile. In the x-direction, the FWHM of the beam expands from 1.49 mm to 4.33 mm as the throughput increases from 30% to 70%. Concurrently, within this throughput range, the beam’s FWHM in the y-direction shifts from 0.61 mm to 1.36 mm.

The primary advantage of throughput modulation lies in its capacity to control beam size. Lower throughputs result in narrower beamlets, which subsequently diminishes the proportion of scattered protons capable of reaching the valleys. This results in a lower valley dose, hence reducing the VPDR and subsequently enhances dose contrast at the entrance. Our simulations found a reduction in VPDR from 0.67 to 0.18 as the throughput was decreased from 70% to 30%.

Exceedingly low throughput, however, can impair the efficiency of the system by wasting a substantial number of protons. In addition, it can significantly decrease the peak dose value, particularly far away from the field center. This reduction is due to the inherent characteristic of the Varian ProBeam system, and most other PBS systems, of delivering divergent beams. With the introduction of thicker collimator septa (lower throughput), lateral proton migration is further inhibited. On the other hand, higher throughput leads to reduced dose contrast (resulting in larger VPDR values) due to the increase in the valley dose. This underlines the balance that must be struck between throughput, efficiency, and dose contrast when optimizing the SFRT dose profile.

## Discussion

**Figure 5:**
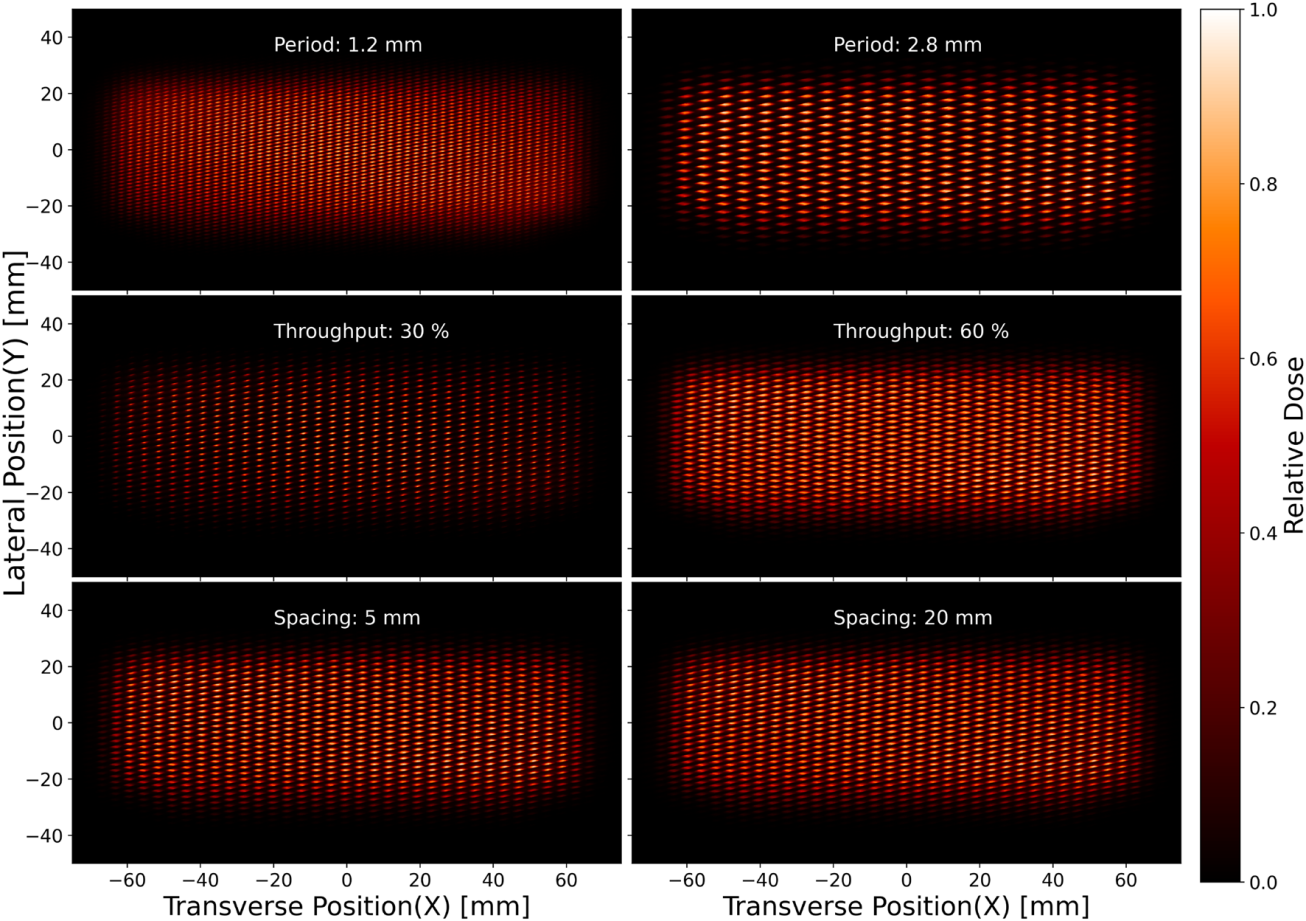
Comparisons of moiré fringes as a function of period, distance between MSCs (spacing), and throughput. Increasing the spacing between the two MSCs has a minor impact on the resulting pattern, but results in slightly less sharp beamlets (bottom two figures).

Our study investigates the potential of utilizing the moiré effect to generate diverse dose profiles for pMBRT using a dual MSC system. The angle between the collimators significantly affects the resulting dose distribution. As the angle varies, the CTC of the dose distribution at the phantom entrance undergoes alterations. Given a specific set of MSC parameters, the dose profile of pMBRT can be tuned simply by adjusting the angle. Our simulations demonstrate the flexibility of such a system for both pre-clinical and clinical application of pMBRT, where differently fractionated dose profiles are desired. Additionally, we have examined how the physical parameters of the MSC can be altered to make further modifications to the fractionated irradiation pattern to make it more suitable for a specific case or tumor. Using the Geant4 Monte Carlo simulation tool, we analyzed the effects of parameters such as period, distance between two MSCs of DCS, and throughput, as illustrated in Fig. 5.

The period of fractionated dose profiles produced by a dual MSC system at various angles was in strong agreement with the theoretical values. This feature of the system provides the opportunity to optimize the angle for a specific tumor in advance. A simple precalibrated rotating system can be installed to adjust the angle during the treatment to deliver the irradiation plan. This will be a very flexible and fast approach to deliver the desired fractionated dose pattern. In addition, a few sets of fixed collimators can be used for further dose adjustment for the entire clinical energy range and a wide range of tumors (shape, size, and location).

Although the magnetic focusing technique could potentially provide a solution for adjusting the pMBRT irradiation profile^33^, it is currently not available in any clinical facility, and its incorporation into existing proton therapy systems would require considerable effort^32^. On the other hand, most current clinical and experimental facilities can, with reasonable effort, incorporate an MSC^57^, thus allowing for the adaptation of the scanning dynamic collimator^32^. However, such a system requires physically repositioning a collimator, which can be a time-consuming process during actual treatment procedures. Furthermore, it is not compatible with a passive scattering system. With these limitations in mind, a DCS emerges as a far more practical concept for tuning fractionated irradiation for radiobiological experiments and clinical applications.

The ability of the dual MSC system to rapidly adjust angles can be particularly advantageous when it is desirable to modify the beam profile based on energy during the irradiation period. Given that the penetration depth of a proton beam into a specific medium is energy-dependent, the broadening of the beam around the Bragg peak due to MCS is also a function of energy. Significant spreads of the beamlets are required to create a lateral overlap to deliver a uniform dose to the PTV. Therefore, a proton beam of higher energy can be employed with a larger CTC distance at the entrance compared to lower-energy beams. In actual treatment plans that employ the SOBP, various energy layers (each with its specific energy spread) are used to cover the entire longitudinal extent of the PTV. The typical time (0.5–1 s) required for a spot scanning system to transition between energies could allow for angle adjustments within the DCS. This flexibility enables dynamic modifications of the irradiation profile, ensuring optimal dose delivery at varying depths of the PTV. This process can further enhance the dose contrast at the entrance (healthy tissue), which is a key requirement in SFRT.

The analytical and simulated moiré periods increased correspondingly as the collimator period increased. Additionally, we noticed a decrease in the VPDR as the collimator period increased, indicating the potential to achieve a more pronounced dose contrast, which is beneficial for SFRT. The influence of collimator throughput on dose profiles was also assessed. Our results showed that varying the throughput while maintaining a constant period allowed for control of the peak dose lateral sizes, directly affecting the dose contrast for a given CTC. Lower throughput resulted in narrower beamlets, which enhanced the dose contrast at the entrance of the phantom. However, excessively low throughputs could impair system efficiency by eliminating a significant number of protons and reducing peak dose values. These observations highlight the necessity of fine-tuning collimator throughput for an optimal balance between dose contrast and system efficiency.

Another captivating feature of the DCS is its ability to fractionate the dose in both lateral directions. This capability could substantially enhance normal tissue sparing compared to a single MSC system. This is achieved through a reduction in the volume of irradiated tissue, taking advantage of dose-volume effects, and thereby potentially reducing the side-effects and improving clinical outcomes. Additionally, with a specific set of collimator parameters established, adjusting the angle does not significantly impact the dose period in the direction orthogonal to the moiré period.

The versatility of DCS extends beyond proton therapy, as it could be compatible with other types of beams, such as photons, electrons, or heavy ions. This compatibility opens up possibilities for incorporation into various SFRT modalities, including GRID or LATTICE therapies. The system’s ability to accurately reproduce varying fractionated dose profiles makes it a viable choice for treatments that require multiple sessions of irradiation.

Finally, it should be mentioned that a periodic moiré pattern may also emerge from the spot-scanning beam delivery and any other structure with lateral periodicity in the beam. This was previously noted for spot-scanning carbon-ion beams in combination with so-called ripple-filters, which are a small range-modulator plate extending the very sharp carbon-ion Bragg peak to a few mm, longitudinally^58^. However, in our simulations presented here, we did not observe an effect stemming from the spot-scanning pattern, even when using a realistic beam model.

## Conclusion

In this study, we investigated the use of the moiré effect to develop a spatially fractionated beam delivery technique for pMBRT (as well as other forms of SFRT in general), relying on a DCS. Our findings provide a comprehensive understanding of the influence of several key MSC parameters on fractionated dose profiles, highlighting the potential of DCS to refine the irradiation profiles of SFRT.

The results obtained using the Geant4 Monte Carlo simulations confirm that the angle between the MSCs of a DCS profoundly affects the dose profile. In particular, we observed a gradual reduction in the CTC from 11.8 mm to 2.4 mm as the angle was increased from 10^∘^ to 50^∘^. We also demonstrated how the physical parameters of the MSC, including the period, throughput, and distance between the MSCs, could be manipulated to fine-tune dose profiles to accommodate specific clinical needs.

A beneficial capability of the DCS is the rapid angle adjustment during PBS energy-layer transitions, allowing for dynamic modifications of the irradiation profile during delivery of a beam. This feature can enhance the dose contrast at various depths, contributing to optimal dose delivery across the target volume. The system’s ability to fractionate the dose in both lateral directions introduces the potential for enhanced tissue sparing by reducing the irradiated volume.

Moreover, the versatility of the dual MSC system extends beyond proton therapy. It is compatible with different beam types and has the potential for integration into various SFRT modalities. The system’s adaptability will likely promote its application in diverse clinical and experimental settings, advancing SFRT strategies.

Our study provides significant insight into the potential of DCS as a tool to advance pMBRT techniques. Its unique potential for dynamic adjustment of irradiation profiles offers a promising avenue for improving patient outcomes in SFRT, ultimately paving the way for further exploration and development in this promising field of cancer treatment.

## Data Availability

The datasets used and/or analyzed during the current study are available from the corresponding author upon reasonable request.
